# Hearing loss among inmates of a juvenile correctional facility in Nigeria

**DOI:** 10.4314/ahs.v22i2.79

**Published:** 2022-06

**Authors:** Habeeb Kayodele Omokanye, Adekunle David Dunmade, Mosunmola Florence Tunde-Ayinmode, Muhammed Mubashir Uthman, Musbau Olawale, Foluwasayo Emmanuel Ologe

**Affiliations:** 1 Department of Otorhinolaryngology, College of Health Sciences, University of Ilorin. PMB 1515, Ilorin, 240003, Nigeria; 2 Department of Behavioural Sciences. College of Health Sciences, University of Ilorin, University of Ilorin Teaching Hospital. Ilorin, Kwara State, Nigeria; 3 Department of Epidemiology and Community Health. College of Health Sciences, University of Ilorin, University of Ilorin Teaching Hospital. Ilorin, Kwara State, Nigeria

**Keywords:** Hearing loss, juvenile offender, audiometry, incarceration, correctional facility, adolescent

## Abstract

**Background:**

Good hearing is essential to learning and rehabilitation of adolescent and young adults in juvenile correctional facilities. Hearing screening programme is not commonly in place for this incarcerated group.

**Objective:**

To evaluate hearing threshold among inmates of a juvenile correctional facility in Nigeria and compare pattern of hearing loss with a control group.

**Methods:**

A total of 135 inmates and equal number of age and sex matched control responded to interviewer-administered questionnaire followed by otoscopy and audiometry.

**Results:**

Mean age of inmates was 19 years ±2.0, while that of control was 18yrs ± 2.5. (p-value 0.077). Four (3%) inmates had bleeding from the ear; otoscopy revealed traumatic tympanic membrane perforation in 2(1.5%) of them. Prevalence of hearing loss was 19.2% and for disabling hearing loss it was 1.4%. Conductive hearing loss was the most common 33(24.4%). Inmates had consistently worse mean hearing thresholds than controls across all frequencies tested in both ears (p-value <0.001).

**Conclusion:**

Hearing loss is prevalent among inmates of juvenile correctional facility. Rehabilitation programme should be balanced with detail attention to health needs of inmates; including pre-admission and periodic hearing screening.

## Introduction

Youths in either conventional schools or rehabilitation homes need good hearing for formal learning^1^–^5^. Otitis media is one of the most common childhood diseases worldwide and is prevalent in developing countries including Nigeria^5^. Many children live with undiagnosed, poorly treated or unaddressed ear diseases till school age; enduring its discomfort and sequel^2^,^5^–^7^. Pre-admission audiometry screening is not a common practice in many schools, hence many children are admitted without a record of their pre-school hearing threshold 7,8. As the population of juvenile and young offenders incarcerated in prisons increases, so is the growing concern about the well-being and increasing health needs of this vulnerable population.^9^–^12^

Although this experience is not peculiar to Nigeria, it is important to provide evidence-based data to guide government policy towards improving health care services in juvenile correctional facilities. This study evaluates hearing health of inmates with a focus on the prevalence, the type and degree of hearing loss among young offenders in one of the three juvenile correctional facilities in Nigeria.

## Methods

One hundred and thirty-five consenting inmates of the juvenile correctional facility were included in the study. Approval was obtained from the ethical review committee of University of Ilorin Teaching Hospital and The Nigerian Prison Service Authority. Security coverage was provided for all our weekly study visits with regulated access to only inmates signed-up for the week. All consented participants responded to interviewer-administered questionnaire. Information obtained included, biodata, socioeconomic background and otological symptoms. This was followed by otoscopy conducted by the author and pure tone audiometry in a quiet room (ambient noise level not exceeding 25 dB (A)) with constant checking of ambient noise level with CRL 412A Testo sound level meter. Pure tone audiometry was performed with a calibrated audiometer (MAICO. MA42 model, with DD45 headphone. Germany) according to recommended standard procedure by British Society of Audiology^13^.

Equal number of age and sex matched boarding students in a conventional Government Secondary School were used as control. Primary outcome measure was four-frequency Pure-Tone Average (4fPTA; average of threshold at 500Hz, 1000Hz, 2000Hz and 4000Hz). Cut off for normal hearing threshold was ≤ 25dB. Level of impairment was classified according to the World Health Organization (WHO) grading^14^. Qualitative type of hearing loss (conductive, sensorineural or mixed) was deduced from audiogram using 10dB air-bone gap for the four frequencies;^15^ tympanometry was not done. Data was analysed using a 2011 IBM SPSS for windows, version 20.0; Armonk, NY. Chi square test was used to compare hearing threshold and other categorical variables in inmates and control. Fisher exact test was applied when expected count in cells was less than five. Level of statistical significance was set at p-value <0.05 at 95% confidence level.

## Results

Mean age of Inmates was 19yrs ±2.0, range 15 – 21 years; while that of Control was 18yrs ± 2.5, range 15 – 21years. There was no statistical difference in the ages of the two groups (p-value =0.077). Frequency of otological complaints was consistently higher among inmates, with a statistically significant difference recorded for hard of hearing (p<0.001), (Table 1). Although otalgia was found to occur more commonly in controls 30(22%) than inmates 21(15.6%), there was no significant difference. (p-value= 0.138). Bleeding from the ear was reported only by inmates (Table: 1); 4(3%) out of whom 2(1.5%) had demonstrable clotted blood on otoscopy (Table 2). Clotted blood was also found in 1(0.7%) of the controls (Table 2).

**Table 1:** Self-Reported Otological Symptoms

Symptoms	Inmates n(%)	Control n(%)	Total	X^2^	P-Value
**Hard of hearing**				21.94	<0.001
**Yes**	43(31.9)	12(8.7)	55(20)		
**No**	92(68.1)	123(91.1)	215(80)		
**Total**	135(100)	135(100)	270(100)		

**Otalgia**					
**Yes**	21(15.6)	30(22)	51(19)	2.20	0.138
**No**	114(84.4)	105(78)	219(81)		
**Total**	135(100)	135(100)	270(100)		

**Otorrhea**				0.68	0.409
**Yes**	15(11)	11(8)	26(10)		
**No**	120(89)	124(92)	244(90)		
**Total**	135(100)	135(100)	270(100)		

**Tinnitus**				4.21	0.040
**Yes**	31(23)	18(13.3)	49(18)		
**No**	104(77)	117(86.7)	221(82)		
**Total**	135(100)	135(100)	270(100)		

**Trauma**				1.65	0.199
**Yes**	15(11)	9(6.9)	24(9)		
**No**	120(89)	126(93.3)	246(91)		
**Total**	135(100)	135(100)	270(100)		

**Bleeding**				4.06	0.044
**Yes**	4(3.0)	0(0.0)	4(1.5)		
**No**	131(97)	135(100)	266(98.5)		
**Total**	135(100)	135(100)	270(100)		

**Table 2:** Otoscopic Findings

	Participants		Statistics		
Otoscopic Findings	Inmate N=135 n(%)	Control N=135 n(%)	Total	X^2^	P-Value
**External Auditory Canal**				2.716	**0.257**
Normal and clear	110(81.5)	119(88.2)	229(84.8)		
Impacted Wax (Cerumen)	18(13.3)	10(7.4)	28(10.4)		
*Others	7(5.2)	6(4.4)	41(4.8)		
**Integrity of TM**					0.005^F^
Intact (Normal)	124(92.0)	134(99.3)	258(95.6)		
Not intact (Perforation or neo-membrane)	11(8.0)	1(0.7)	12(4.4)		
**TM light reflex**				5.208	0.023
Both TM shining (Normal)	100(74.0)	116(86.0)	216(80.0)		
Both or one TM Dull	35(26.0)	19(14.0)	54(20)		
**Position of TM**					< .001^F^
Both TM Normal (No retraction)	118(87.4)	134(99.3)	252(93.3)		
Both or one TM retracted	17(12.6)	1(0.7)	18(6.6)		

F Fisher exact test.

* Others represent; desquamated debris, active discharge, foreign body (cotton bud), clotted blood, fungal debris.

Cerumen (wax) was the most common content of the external auditory canal (EAC); seen in 18(13.3%) of inmates and 10(7.4%) of controls. Almost all participants in the control group, 134(99.3%) had normal TM integrity as against 124(92.0%) of the inmates with either a demonstrable TM perforation or neomembrane; with a significant statistical difference; (p-value = 0.005), (Table: 2). Dull T.M was more common in inmates 35(26%) than the control 19(14.1%); p-value = 0.037. Retracted TM occurs more commonly in inmates 17(12.6%) than the control 1(0.7%); fisher exact = 0.001. (Table: 2).

**Fig. 1:**
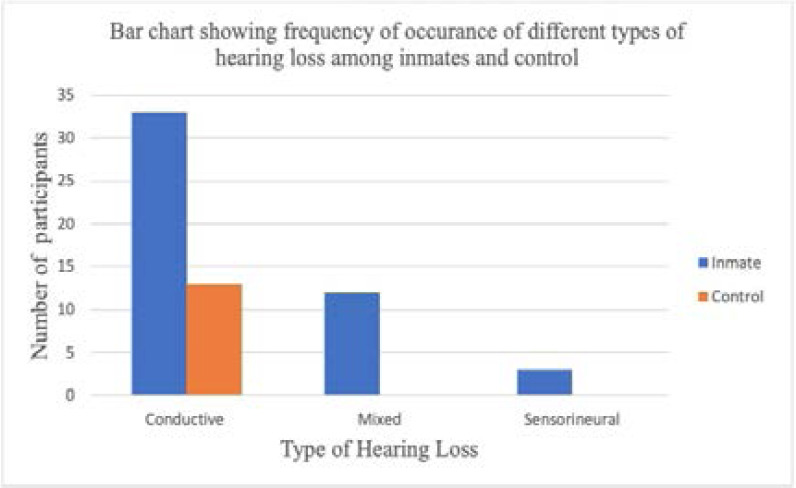
Types of hearing loss

Conductive hearing loss was most common among inmates 33(24.4%) than control 13(9.4%) and it was the only type of hearing loss seen among controls. Sensorineural hearing loss (SNHL) and mixed hearing loss (MHL) constituted 3(2.2%) and 12(9.0%) respectively among inmates (Fig. 1).

Prevalence of hearing loss was 19.2% in the better and 34.8% in the worse ear of inmates. While the prevalence of disabling hearing, loss was 1.4% in the better and 6.6% in the worse ear of inmates. Hearing was normal in the better ears of controls, while the prevalence of hearing loss in the worse ear of control was 2.2% (Table 3).

**Table 3:** Air Conduction Hearing in the Worse and Better Ear on Pure Tone Audiometry

	Participants		
	Inmates N=135 n(%)	Control N=135 n(%)	Total
**Hearing level**			
**Better Ear**			
Normal (25 dB HL or less)	109(80.7)	135(100)	244(90.3)
Slight (26–40 dB HL)	24(17.9)	0(0.0)	24(8.9)
Moderate (41–60 dB HL)	1(0.7)	0(0.0)	1(0.4)
Severe (6 1–80 dB HL)	0(0.0)	0(0.0)	0(0.0)
Profound (81 dB HL or greater)	1(0.7)	0(0.0)	1(0.4)
**Worse Ear**			
Normal (25 dB HL or less)	87(64.5)	122(90.4)	209(77.4)
Slight (26–40 dB HL)	39(28.9)	10(7.4)	49(81.1)
Moderate (41–60 dB HL)	6(4.4)	0(0.0)	6(2.2)
Severe (6 1–80 dB HL)	2(1.5)	3(2.2)	5(1.9)
Profound (81 dB HL or greater)	1(0.7)	0(0.0)	1(0.4)

Of the 26 (19.2%) inmates with suboptimal hearing in their better ears; 24(17.9%) had slight, 1(0.7%) each had moderate or profound impairment. (Table 3). Whereas, in the worse ear of inmates, hearing was impaired slightly in 39(28.9%), moderately in 6(4.4%), severe in 2(1.5%) and profound in 1(0.7%). Profound hearing loss was seen only in the worse ears of inmates while slight 10(7.4%) and severe 3(2.2%) degree of hearing loss were the type seen in the worse ears of control. (p-value<0.001) (Table 3).

**Fig. 2:**
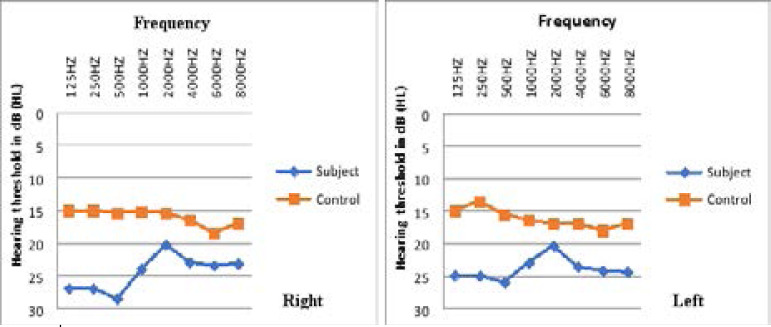
Mean air conduction hearing level Vs frequency; on the right and left ear of subjects and control

There was a consistently higher mean hearing threshold in both ears of inmates than controls across all frequencies tested. (Fig. 2).

## Discussion

By reasons of incarceration, inmates in juvenile correctional facility are often deprived the benefits of contact with the larger society including access to medical care^16^,^17^. This portends low access to healthcare services with higher risk of ear diseases being undiagnosed or poorly treated. Hence, incarcerated youths face disproportionately higher morbidity and mortality compared to the general adolescent population^18^. Although, a generally low patronage of otolarygological services exists in our setting due to non-awareness among parents, poverty and illiteracy^19^; parental neglect of children is also a likely reason for higher risk of untreated ear diseases among inmates compared with their counterparts in conventional schools^20^–^22^.

More inmates were found with untreated impacted wax than controls (Table 2). On the other hand, foreign body (cotton bud) found in equal frequency among imates and control reflects the generally common practice of self ear cleaning with cotton bud, thus predisposing to foreign body in the ear. However, none of those with foreign body in the ear had neither associated bleeding nor blood clot in the EAC. Two of the four inmates with complaint of bleeding from the ear gave history of receiving frequent slaps on the face, and clotted blood was found in their EACs on otoscopy (Table 1 and 2). Hence, the combination of perforations (persistent or healed) and blood clot observed more in the EACs of inmates may be stigmata of abuse and battering. Earlier report on child rights in Nigeria documented that more than half of juvenile and young adults experienced verbal abuse and physical assault by police or warder during arrest or detention^23^. It has been proven that compressional injury with TM rupture can occur from a blow or slap on the ear, delivered at a pressure of up to 25 pounds per square inch with simultaneous occlusion of the external auditory canal^23^. It is usually accomplished by hearing loss, pain, feeling of fullness and slight bleed^24^.

Otalgia was not a prominent symptom among inmates (Table I) and acute middle ear infection was seen only among controls; while otoscopic findings in inmates were in keeping with chronic untreated middle and external (fungal) ear infections. Similarly, thickened TM, neomembrane and TM perforation were indicative of long standing inflammatory process and chronicity of ear disease. This finding correlated reasonably well with aetiological diagnosis on otoscopy. Conductive hearing loss being the most common among inmates suggests treatable causes of hearing loss with potentially good prognosis using medical and/or surgical intervention^25^. More so, preventive measures could be targeted at risk factors like overcrowded condition and poor hygiene^26^. Early diagnosis and treatment would prevent detrimental effect of hearing loss on social interaction and socio-economic attainment of those individuals after re-integration into the society.

Mean hearing threshold of inmate was worse than that of control across all frequencies (Fig. 2). This is consistent with findings by Jacobson in a study of 34 penitentiary prison inmates, all with previous drug use^27^. Synergistic effect of drug abuse, noise exposure and head trauma were identified as possible contributory factors. Although, our findings of mixed and sensorineural hearing loss among only inmates were not investigated further in this study, many of the inmates admit exposure to illicit substances some of which may have contributory ototoxic effects. The most significant hearing risk behaviour found among young offenders in an earlier study was listening to loud music with the headphones^7^. Noise induced hearing loss will often manifest as sensorineural hearing loss, this study did not investigate drug use or noise exposure among participants in relation to hearing loss.

Prevalence of hearing loss was 19.2% in the better and 34.8% in the worse ear of inmates in this study. However, slight degree of hearing loss was the most common. This degree of hearing loss could go unnoticed by parent or teacher because it develops slowly over years that lip-reading could compensate for the deficit. Indig et al. in a baseline health survey of young people in custody in New South Wales quoted a prevalence of 18% for mild to moderate hearing loss in one ear or both ears with 32% having at least one ear having a degree of hearing loss^7^. In the same study 50% of inmates had normal hearing in both ears as against 68.1% of inmates and 91.1% of control in this study. A review of violence in deaf and hard-of-hearing people revealed a huge presence of hearing-impaired people among prison population; in keeping with a vicious cycle of violence provoked by frustrations created by worsening hearing loss^28^. In a study of the link between hearing impairment in early childhood and youth offending in Aboriginal children, He et al. confirms evidence for an association between hearing impairment and youth offending, for boys only.^29^ Although, the population in that study was much younger than ours, we found a prevalence of disabling hearing loss of 1.4% and 6.6% in the better and worse ear of inmates respectively and 2.2% in the worse ear of control. Prevention or correction of such a disability could improve the outcome of rehabilitation in juvenile prisons.

Hearing loss is prevalent among inmates of juvenile prison. It is often of mild degree, and progression may be preventable by early diagnosis and treatment. Rehabilitation programme in the juvenile correctional facilities should be balanced with detail attention to health needs of inmates; including baseline and periodic hearing screening and prompt access to hearing heath care.
